# Supplementary material for: Lutein Decreases Inflammation and Oxidative Stress and Prevents Iron Accumulation and Lipid Peroxidation at Glutamate-Induced Neurotoxicity
Source: Antioxidants (Basel). 2022 Nov 17;11(11):2269. doi: 10.3390/antiox11112269 (PMC9687421; doi:10.3390/antiox11112269)
Supplement: Supplementary file 1 [file antioxidants-11-02269-s001.zip › antioxidants-2034926-supplementary.pdf]

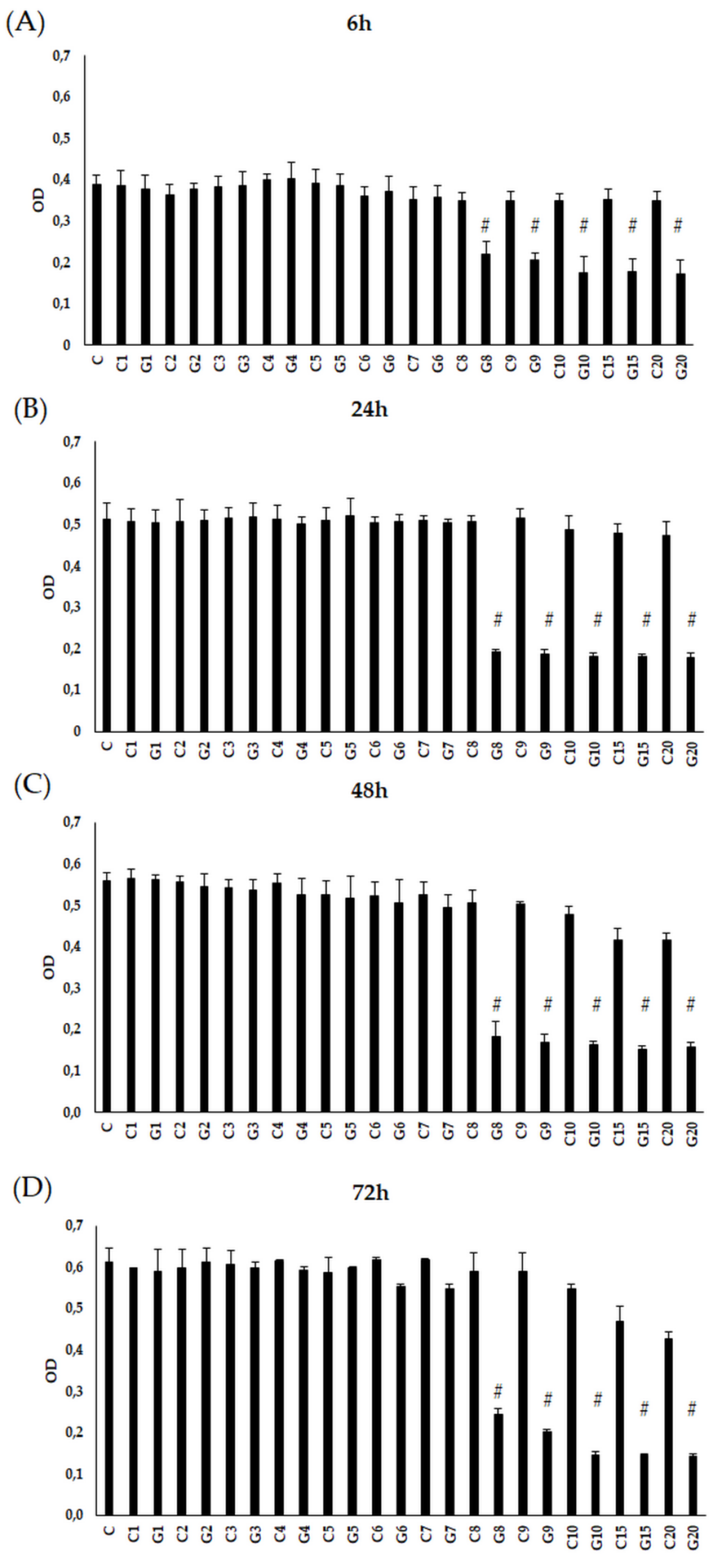

(A)
6h
OD
0,7
0,6
0,5
0,4
0,3
0,2
0,1
0
#
C C1 G1 C2 G2 C3 G3 C4 G4 C5 G5 C6 G6 C7 G6 C8 G8 C9 G9 C10 G10 C15 G15 C20 G20
(B)
24h
OD
0,7
0,6
0,5
0,4
0,3
0,2
0,1
0
#
C C1 G1 C2 G2 C3 G3 C4 G4 C5 G5 C6 G6 C7 G7 C8 G8 C9 G9 C10 G10 C15 G15 C20 G20
(C)
48h
OD
0,7
0,6
0,5
0,4
0,3
0,2
0,1
0,0
#
C C1 G1 C2 G2 C3 G3 C4 G4 C5 G5 C6 G6 C7 G7 C8 G8 C9 G9 C10 G10 C15 G15 C20 G20
(D)
72h
OD
0,7
0,6
0,5
0,4
0,3
0,2
0,1
0,0
#
C C1 G1 C2 G2 C3 G3 C4 G4 C5 G5 C6 G6 C7 G7 C8 G8 C9 G9 C10 G10 C15 G15 C20 G20

(E)

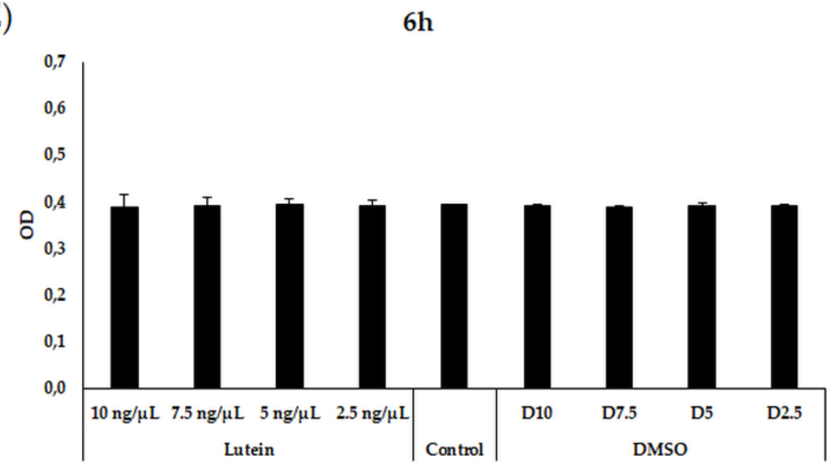
6h
0,7
0,6
0,5
0,4
0,3
0,2
0,1
0,0
OD
10 ng/µL
7.5 ng/µL
5 ng/µL
2.5 ng/µL
Lutein
Control
D10
D7.5
D5
D2.5
DMSO

(F)

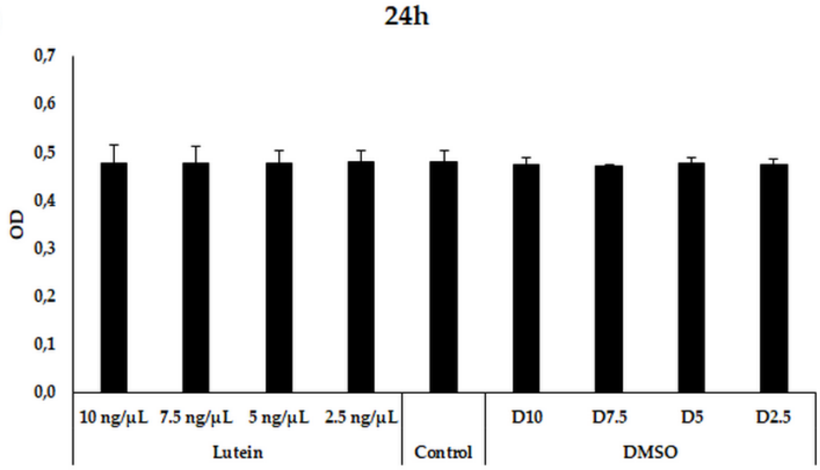
24h
0,7
0,6
0,5
0,4
0,3
0,2
0,1
0,0
OD
10 ng/µL
7.5 ng/µL
5 ng/µL
2.5 ng/µL
Lutein
Control
D10
D7.5
D5
D2.5
DMSO

(G)

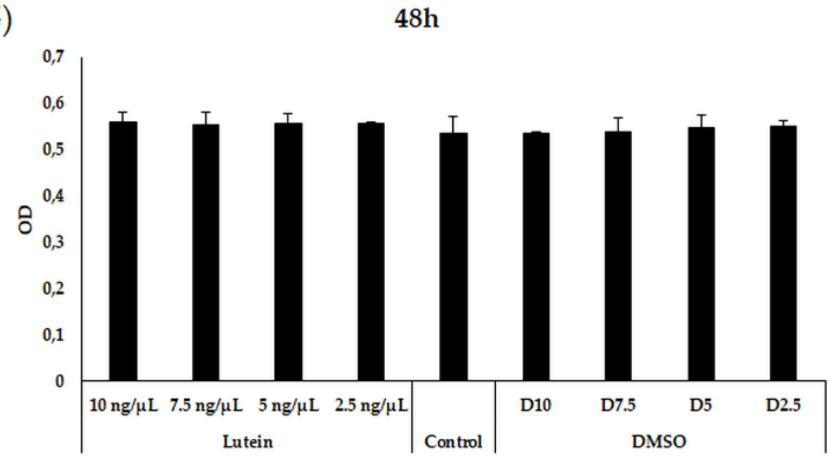
48h
0,7
0,6
0,5
0,4
0,3
0,2
0,1
0
OD
10 ng/µL
7.5 ng/µL
5 ng/µL
2.5 ng/µL
Lutein
Control
D10
D7.5
D5
D2.5
DMSO

(H)

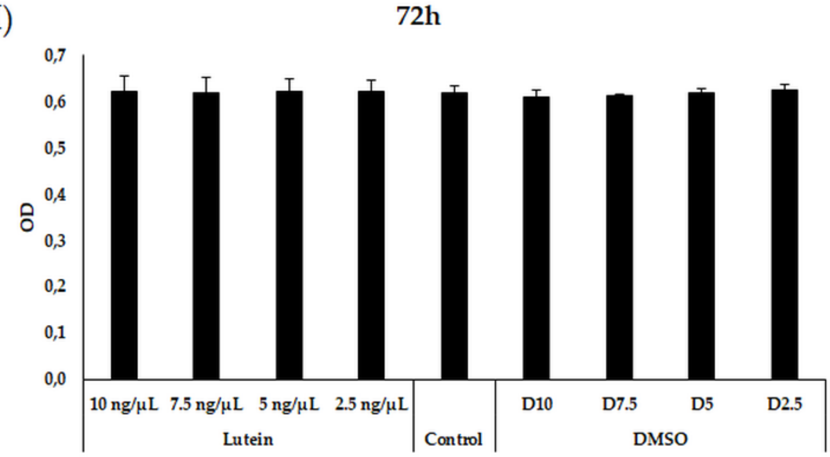
72h
0,7
0,6
0,5
0,4
0,3
0,2
0,1
0,0
OD
10 ng/µL
7.5 ng/µL
5 ng/µL
2.5 ng/µL
Lutein
Control
D10
D7.5
D5
D2.5
DMSO

(I)

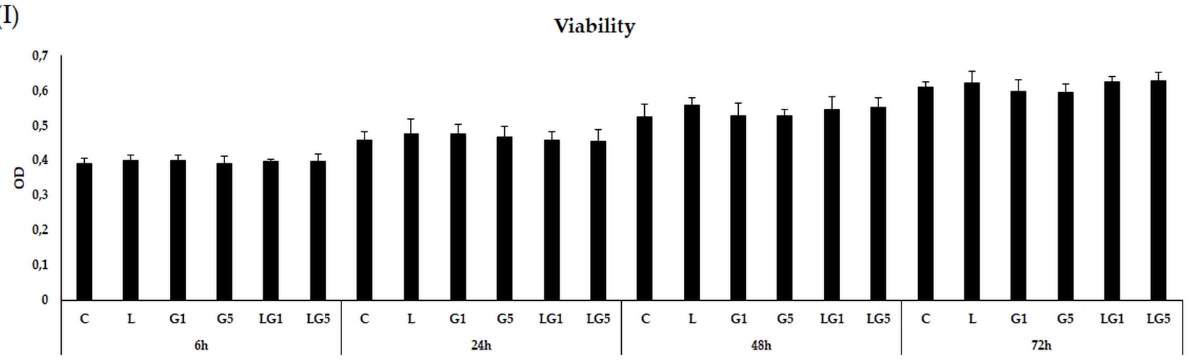

Figure S1. Cell viability determinations of the lutein and glutamate-treated SH-SY5Y cells. Viability was measured using a resazurin-based cell viability assay after lutein and/or glutamate treatments. The mean optical density of the wells indicates the viable cells from four independent experiments, each measured in triplicate.

Viability is expressed as the percentile of the total cell number of the untreated control cells, in the case of lutein as a percentile of the total cell number of the appropriate DMSO-treated control cells. (**A**) Cell viability of glutamate and DMSO-treated SH-SY5Y cells at 6 h. (**B**) Cell viability of glutamate and DMSO-treated SH-SY5Y cells at 24 h. (**C**) Cell viability of glutamate and DMSO-treated SH-SY5Y cells at 48 h. (**D**) Cell viability of glutamate and DMSO-treated SH-SY5Y cells at 72 h. (**E**) Cell viability of lutein and DMSO-treated SH-SY5Y cells at 6 h. (**F**) Cell viability of lutein and DMSO-treated SH-SY5Y cells at 24 h. (**G**) Cell viability of glutamate and DMSO-treated SH-SY5Y cells at 48 h. (**H**) Cell viability of glutamate and DMSO-treated SH-SY5Y cells at 72 h. (**I**) Cell viability of lutein and/or glutamate-treated SH-SY5Y cells at 6 h – 72 h. Cell viability assays were made in triplicate in four independent experiments. The bars represent mean values and error bars represent the standard deviation (SD) for four independent experiments (n=4). The * indicates $p < 0.05$ compared to the appropriate DMSO control at 6 h, 24 h, 48 h or 72 h. # signs the statistical significance of combined (lutein with Glu) treatments compared to the Glu treatments at 24 h, 48 h, and 72 h ($p < 0.05$). Abbreviations of treatments: C-absolute control; DMSO controls: D2.5-DMSO equivalent to 2.5 ng/μL of lutein; D5-DMSO equivalent to 5 ng/μL of lutein; D7.5-DMSO equivalent to 7.5 ng/μL of lutein; D10-DMSO equivalent to 10 ng/μL of lutein; C1-DMSO equivalent to 1 mM glutamate; C2-DMSO equivalent to 2 mM glutamate; C3-DMSO equivalent to 3 mM glutamate; C4-DMSO equivalent to 4 mM glutamate; C5-DMSO equivalent to 5 mM glutamate; C6-DMSO equivalent to 6 mM glutamate; C7-DMSO equivalent to 7 mM glutamate; C8-DMSO equivalent to 8 mM glutamate; C9-DMSO equivalent to 9 mM glutamate; C10-DMSO equivalent to 10 mM glutamate; C15-DMSO equivalent to 15 mM glutamate; C20-DMSO equivalent to 20 mM glutamate; glutamate treatments: G1- 1 mM glutamate; G2- 2 mM glutamate; G3- 3 mM glutamate; G4- 4 mM glutamate; G5- 5 mM glutamate; G6- 6 mM glutamate; G7- 7 mM glutamate; G8- 8mM glutamate; G9- 9 mM glutamate; G10- 10 mM glutamate; G15- 15 mM glutamate; G20- 20 mM glutamate.
